# Review: Systematic review and meta‐analysis – financial incentives increase engagement with parenting programs for disruptive behavior problems

**DOI:** 10.1111/camh.12746

**Published:** 2024-12-21

**Authors:** Nathan Hodson, Madiha Majid, Richard James, Eileen K. Graham, Daniel K. Mroczek, Rinad S. Beidas

**Affiliations:** ^1^ Unit of Mental Health and Wellbeing, Warwick Medical School University of Warwick Coventry UK; ^2^ Coventry and Warwickshire Partnership NHS Trust Coventry UK; ^3^ Nemours Children's Hospital Wilmington DE USA; ^4^ Department of Medical Social Sciences Northwestern University Feinberg School of Medicine Chicago IL USA; ^5^ Department of Psychology Weinberg College of Arts & Sciences, Northwestern University Evanston IL USA

**Keywords:** Meta‐analysis, parenting training, parenting, conduct disorder, disruptive behavior

## Abstract

**Background:**

To evaluate the effect of financial incentives on engagement in parenting programs for disruptive behavior disorders, as well as effect on child behavior. As a secondary aim, demographic differences were investigated as effect modifiers.

**Methods:**

We searched PubMed, CINAHL, Sociological Abstracts, Cochrane Trials, and PsycINFO for randomized controlled trials and quasi experimental studies offering parents a financial incentive for engagement with parenting programs targeting disruptive behavior in children aged under 18, vs no incentive. Engagement in each group was evaluated at four stages: connection, attendance, participation, and enaction. Per protocol (CRD42022336210) random effects meta‐analysis was conducted using Stata‐16. Meta‐analyses of binary data used a log odds ratio and continuous data was standardized using Hedges' *g*.

**Results:**

We identified 2438 papers and screened 35 at full length. We included eight independent cohorts from seven papers. Parents invited to incentive arms were more likely to complete a threshold of sessions than parents invited to control arms (odds ratio 2.51 95% CI 1.42–4.48). Parents were more likely to agree to participate when they knew they were joining the incentive program (odds ratio 1.40, 95% CI 1.20–1.65) and parents in the incentive group were more likely than parents in the control group to reach a completion threshold of sessions (odds ratio 1.76 95% CI 1.17–2.66).

**Conclusion:**

Incentives increase parenting programs engagement among parents who are invited and among parents who have begun attending programs. Incentives are an effective potential tool for increasing engagement but further research is needed to establish acceptability and optimal design.

**Key Practitioner Message**

**What is known?**

- Promoting parental engagement with programs targeting disruptive behavior disorders is an ongoing challenge. Small financial incentives have improved engagement in other areas of healthcare

**What is new?**

- This is the first systematic review and meta‐analysis to explore whether financial incentives increase engagement in parenting programs targeting disruptive behavior disorders. Our meta‐analysis finds that financial incentives increase the number of parents enrolled in programs and increase the proportion of parents reaching a threshold of sessions.

**What is significant for clinical practice?**

- Small financial incentives provide a potential tool for increasing engagement among high‐risk and hard‐to‐reach families. Services may consider implementing a range of incentives to evaluate local effectiveness and acceptability.

## Introduction

Evidence‐based parenting programs are the recommended first line for disruptive behavior disorders such as oppositional defiant disorder (ODD), conduct disorder (CD), and attention deficit/hyperactivity disorder (ADHD) (NICE, 2013, 2019). These programs support parents to use techniques that encourage children's positive behaviors and reduce disruptive behavior, but low levels of program completion mean many families do not experience these benefits (Chacko et al., 2016; Furlong et al., 2012; NICE, 2013, 2019). Evidence suggests that financial incentives may increase engagement across diverse health behaviors (Vlaev, King, Darzi, & Dolan, 2019).

Disruptive behavior disorders are among the most common mental health conditions of childhood, affecting around 5% of children globally (Diagnostic and statistical manual of mental disorders, 2013; Fairchild et al., 2019; Ghandour et al., 2019; Green, Meltzer, Ford, & Goodman, 2005). Oppositional defiant disorder (ODD) is characterized by anger, argumentativeness, and vindictiveness, and conduct disorder (CD) is characterized by persistent violations of the rights and boundaries of adults and other children. In attention deficit/hyperactivity disorder (ADHD), the core symptoms are inattention or hyperactivity‐impulsivity (Faraone et al., 2015). Untreated, long‐term outcomes for children with disruptive behavior disorders are poor, including higher risk of drug misuse, failure to thrive educationally, and contact with the criminal justice service (Caspi et al., 1997; Faraone et al., 2015; Fergusson, Horwood, & Ridder, 2005). Over a lifetime, these problems are associated with high costs to the individuals and families who experience them, as well as societal costs (Gardner et al., 2017).

Parenting programs involve a range of different modes and typically take place over several sessions, including learning through discussions, roleplay and homework. As parenting styles change as a result of these programs, maladaptive behavior cycles are broken, child behavior problems are reduced, and long‐term outcomes are improved (Garland, Hawley, Brookman‐Frazee, & Hurlburt, 2008; Sandler, Schoenfelder, Wolchik, & MacKinnon, 2011). However, many families do not have the opportunity to benefit: a 2016 systematic review found that only 49% of referred parents complete these programs and 25% do not initiate them after referral (Chacko et al., 2016). There are several stages to effective engagement with parenting programs. Piotrowska et al.'s Connect, Attend, Participate, Enact (CAPE) model (2017) describes four stages to parenting program engagement: ‘connect’—initial registration with a program, ‘attend’—physical presence at sessions, ‘participate’—interaction with session activities and content, and ‘enact’—change in parenting style.

Several interventions have been designed to improve all four stages of engagement, including resource‐intensive approaches such as motivational interviews and brief interventions building on structural family therapy (Florean, Dobrean, Pasarelu, Georgescu, & Milea, 2020; Ingoldsby, 2010). One study evaluated whether motivational interviewing could support ongoing attendance and participation in parenting programs and found a 15% increase in engagement compared with treatment as usual (Nock & Kazdin, 2005), although other studies using motivational interviewing to change parenting behavior in vulnerable families have found no difference (Mullins, Suarez, Ondersma, & Page, 2004). Motivational interviewing is a resource‐intensive approach, and cost‐effectiveness studies in similar contexts have had inconclusive results (Doring et al., 2018; Ingoldsby, 2010). This approach costs hundreds of dollars per parent, whether or not engagement increases, so it may not be the most effective use of resources (Doring et al., 2018).

Online delivery of parenting programs may provide a less resource‐intensive means of increasing connection and attendance by reducing the need for transport and childcare, as well as potentially reducing the stigma of attending a parenting group (Florean et al., 2020; Ingoldsby, 2010). However, online parenting programs generally lack the social benefits of groups which promote participation (Florean et al., 2020). In the largest study of an online parenting program to date only 7% of referred parents completed the program (Dadds et al., 2019), suggesting poorer engagement than the average face‐to‐face program where roughly one in three referred parents completes the program (Chacko et al., 2016). These problems are particularly marked among ethnic and racial minoritized communities and among people from economically disadvantaged backgrounds (Cohen et al., 2016; O'Byrne, Thompson, Friedmann, & Lumley, 2022). Despite existing approaches to increase engagement, many families continue to miss out on parenting programs.

Financial incentives can offer a relatively low‐cost and scalable approach to changing behavior (Vlaev et al., 2019). Financial incentives have increased healthy behaviors in diverse healthcare settings including vaccinations, HIV‐testing, weight loss, and antipsychotic treatment adherence (Adams, 2021; Hodson et al., 2022; Priebe et al., 2013; Thirumurthy et al., 2019; Vlaev et al., 2019). Public health policy initiatives, including mental health, increasingly incorporate financial incentives (Board, 2020; Hodson, 2021). Under these arrangements participants receive financial compensation upon completing a healthy behavior (e.g., an example or two). This offer may be a lottery or may be a single payment (Law, Peterson, Walkey, & Bosch, 2022). It may be gain‐framed (where participants stand to receive money) or loss‐framed (where they risk losing money) (Hodson, 2022). Incentives like these may change health behavior, have no effect, or even decrease healthy behaviors (Judah et al., 2018). A 2010 systematic review (Ingoldsby, 2010) compared initiatives to increase parenting program attendance and identified three studies of financial incentives but did not describe the populations, incentive designs, or effect sizes in sufficient detail to draw conclusions but it is plausible that the connection, attendance, participation and enaction stages of engagement could all be affected by financial incentives. Given the urgent need to increase engagement with parenting programs and the known effect of financial incentives on engagement elsewhere in healthcare, it is important to review the literature into the effect of financial incentives on engagement with parenting programs. This systematic review explores whether financial incentives can increase parenting programs engagement.

### Aims

This systematic review and meta‐analysis evaluated the effect of financial incentives on engagement in parenting programs across the four stages of engagement using the CAPE model of engagement—connect, attend, participate, and enaction—as well as any reported effect on child behavior (Piotrowska et al., 2017). As a secondary aim, demographic differences in response to financial incentives were also investigated (BeLue, Halgunseth, Abiero, & Bediako, 2015). Prespecified review questions are given below.

1. Do financial incentives for engagement in parenting programs affect parental engagement as defined by the ‘Connect, Attend, Participate, Enact’ model, where ‘enact’ refers to changes in parenting style (measured using tools such as the Parenting Scale (Arnold, O'Leary, Wolff, & Acker, 1993) or the Alabama Parenting Scale (Shelton, Frick, & Wootton, 1996))?
2. Do financial incentives for engagement in parenting programs lead to increased engagement among groups experiencing structural inequities, including racism, discrimination, and poverty?
3. Have outcomes of financial incentives differed based on their design; for example, the size of the incentives, whether they are certain or uncertain, and whether they use cash or vouchers?
4. As a secondary consideration, do financial incentives for engagement at parenting programs lead to improved child behavior?

We define structural inequities as ‘the systemic disadvantage of one social group compared to other groups with whom they coexist, and the term encompasses policy, law, governance, and culture and refers to race, ethnicity, gender or gender identity, class, sexual orientation, and other domains’ (The Root Causes of Health Inequity, 2017).

## Methods

This systematic review was conducted in line with the Preferred Reporting Items for Systematic Reviews and Meta‐Analysis (PRISMA) guidelines (Liberati et al., 2009). The protocol was registered in PROSPERO (CRF42022336210) and published (Hodson, Majid, James, et al., 2023).

### Eligibility criteria

Eligibility criteria were set out in our published protocol (Hodson, Majid, James, et al., 2023).

#### Participants

Studies were included if the participants were guardians of children aged up to 18 years old and concerns about children's behavior had been identified by parents or by professionals. There were no limitations on behavior problem severity or diagnosis of ODD, CD, and/or ADHD given parenting programs can improve subclinical or severe behavior problems and can be used preventively (Furlong et al., 2012). In clinical practice many children with suspected ODD or CD are not formally diagnosed given reluctance to label children and, as such, referral to parenting skills programs is not restricted to parents of children with a formal diagnosis but tends to include parents who have concerns about their children's behavior and parents who have been referred due to professionals' concerns about their children's behavior. Therefore, experimental research into the impact of financial incentives in the context of parenting programs has not restricted participation to the parents of children with a formal diagnosis. In keeping with the prevailing approach in the literature, we included any parents who had been referred or had self‐referred to a parenting program due to concerns about their child's behavior. There was no restriction on family composition. There were no limitations on parent characteristics or demographics.

#### Intervention

Studies were included if they offered any parenting program intended to improve child behavior problems with at least 2 sessions, whether face‐to‐face or online, individual or group‐based. There were no restrictions on setting. There were no restrictions on specific intervention content or pedagogic approach.

Studies were included if parents (not children) were offered a financial incentive conditional upon completing one of the stages of the CAPE model of engagement. Financial incentives were defined as any financial payment conditional upon completion of some action where the recipient has prior awareness of the arrangement. There was no restriction on incentive magnitude or form (e.g., cash, discounts, vouchers) but studies only offering benefits in‐kind (e.g., meals, transport, childcare) were excluded. Lotteries and guaranteed incentives and gain‐framed and loss‐framed incentives were included (Vlaev et al., 2019). Conditional cash transfer studies were only included if parenting skills training was the only condition for the cash transfer; the study was not included if additional public health or education conditions such as attending training on healthy eating were included.

#### Comparators

Included studies compared parents who received financial incentives with those who did not.

#### Outcomes

Measures of engagement covered all stages of the CAPE model. Connect, defined as reach (calculated as number of participants divided by number of people invited). Attend, defined as physical attendance at group sessions, which was calculated in two ways: (a) the number of participants who reached a threshold for completing the program divided by the total number of participants; (b) the mean of the proportion of available sessions completed by each participant. Participate, defined as any measures of homework completion or discussion (Piotrowska et al., 2017). Enact, defined as parenting style change and collected via parental behavior‐change measures. Finally, secondary outcomes of interest related to child behavior and included the Eyberg Child Behavior Inventory (ECBI) (Gross et al., 2007), Child Behavior Checklist (CBCL) (Mansolf, Blackwell, Cummings, Choi, & Cella, 2022) and SDQ conduct scale (Goodman, 2001).

#### Types of studies

Included studies were randomized controlled trials or quasi‐experimental studies (i.e., studies with a control group allocated through a nonrandom process).

### Search strategy

Searches were designed in partnership with a biomedical research librarian (RJ) and core team members (NH and RB). The following databases were searched: PubMed, CINAHL (EBSCO), Sociological Abstracts (ProQuest), Cochrane Trials, and PsycINFO (ProQuest). Searches were initially conducted in April 2022 and updated on 1st September 2023. Search terms included subject headings and text words and phrases related to financial incentives, and to parent training, families, and parent–child relationships (see Appendix S1). Forward and backward citation chaining was conducted to screen for additional studies.

No new translations were undertaken so non‐English language papers were excluded. Papers published prior to 1970 were excluded. There were no restrictions by country or clinical setting.

### Study selection process

Results were exported into Covidence systematic review software (Veritas Health Innovation, Melbourne, Australia, 2023) and duplicates removed. The abstracts and titles of the remaining papers were screened against the inclusion criteria by two independent reviewers (NH and MM). Potentially relevant papers were assessed at full text by two independent reviewers (NH and MM). No disagreements requiring a third reviewer arose.

### Assessment of methodological quality

Two reviewers critically appraised eligible studies using the JBI checklists for Randomized Controlled Trials and for Quasi‐Experimental Studies (JBI, 2020a, 2020b). Missing data was obtained from original study authors. No disagreements requiring discussion between RB, NH and MM arose. The methodological quality of each included study was illustrated with tables.

### Data collection process

Data was extracted from the papers using Covidence following pilot testing of a data extraction form by NH and MM. CONSORT flow diagrams for each study were reviewed to measure reach, which represents the ‘Connect’ stage of engagement. Where CONSORT flow diagrams were not presented, the text was searched for these details. Reviewers recorded whether potential participants were aware of which arm they would be allocated to. Session ‘Attendance’ was extracted by number of participants, number completing all modules, and mean number of modules completed by participants. Any threshold used for treating attendance as a binary was also extracted. Attendance data in other forms was also extracted.

To determine ‘Participation’, data on program ‘homework’ completion and any others measures of participation (e.g., leader evaluation of parent participation) was extracted. Change in parenting style (the ‘Enact’ stage) was measured using validated instruments, where possible. Any other observer‐rated measure of change in parenting style were also extracted.

A secondary endpoint was child‐behavior problems. Pre‐ and postmeasures of child behavior problems in control and intervention arms were extracted. Any validated scales of disruptive behavior, such as Eyberg Child Behavior Inventory (Gross et al., 2007), Child Behavior Checklist Externalizing Scale (Mansolf et al., 2022), Strengths and Difficulties Questionnaire (Goodman, 2001).

Demographic data on trial populations was extracted, including parent and child age, sex, socioeconomic status, education level, mental health status, incarceration status, immigration status, marital status, ethnicity, and race. Intervention setting (e.g., university, clinic, community) was also extracted.

### Data synthesis

Results are reported descriptively, including details of cohorts and financial incentive regimes. Financial incentive magnitudes were standardized in two steps: first (if required) they were converted to USD at the exchange rate of the date of publication, then they were updated to account for inflation and standardized for June 2024 (CPI Inflation Calculator, 2024; European Central Bank, 2023).

Meta‐analysis was also conducted per protocol, because data for at least three studies was available. Control and incentive groups were compared regarding first, proportion of participants who completed programs, second, proportion of invited parents who agreed to participate third, proportion of invited parents who completed the program and fourth, mean number of modules completed.

Meta‐analysis code for Stata‐16 was prepublished in the protocol. We used a random effects model given differences in incentive designs and cohorts could create real differences in the effect of the incentive. Meta‐analyses of binary data used a log odds ratio and continuous data was standardized using Hedges' *g* (Borenstein, Hedges, Higgins, & Rothstein, 2010). Results were reported graphically using forest plots. Risk of bias was assessed using funnel plots where appropriate. Heterogeneity was assessed with *I* ^2^ and Cochran's *Q* (Cochran, 1954). Log odds ratios were converted back into odds ratios for ease of presentation and both odds ratios and log odds ratios were reported.

Results were interpreted in the context of cohort characteristics, incentive regimes, and the literature on behavioral economics and financial incentives. Tables were created to show how financial incentive designs related to effect sizes.

### Assessing certainty in the findings

A Summary of Findings was created following the Grading of Recommendations, Assessment, Development and Evaluation (GRADE) approach for assessing the quality of evidence (Guyatt et al., 2008). The outcomes reported in the Summary of Findings included mean number of modules completed, participant completion, and odds ratio of participants completing the program. Each outcome was assigned a ranking of high, moderate, low, or very low regarding the quality of evidence based on the risk of bias. The rationale behind each GRADE evaluation addresses the topics given in the Cochrane training handbook (Schünemann et al., 2022) and was reported in Appendix S10.

## Results

### Study selection

**Figure 1:**
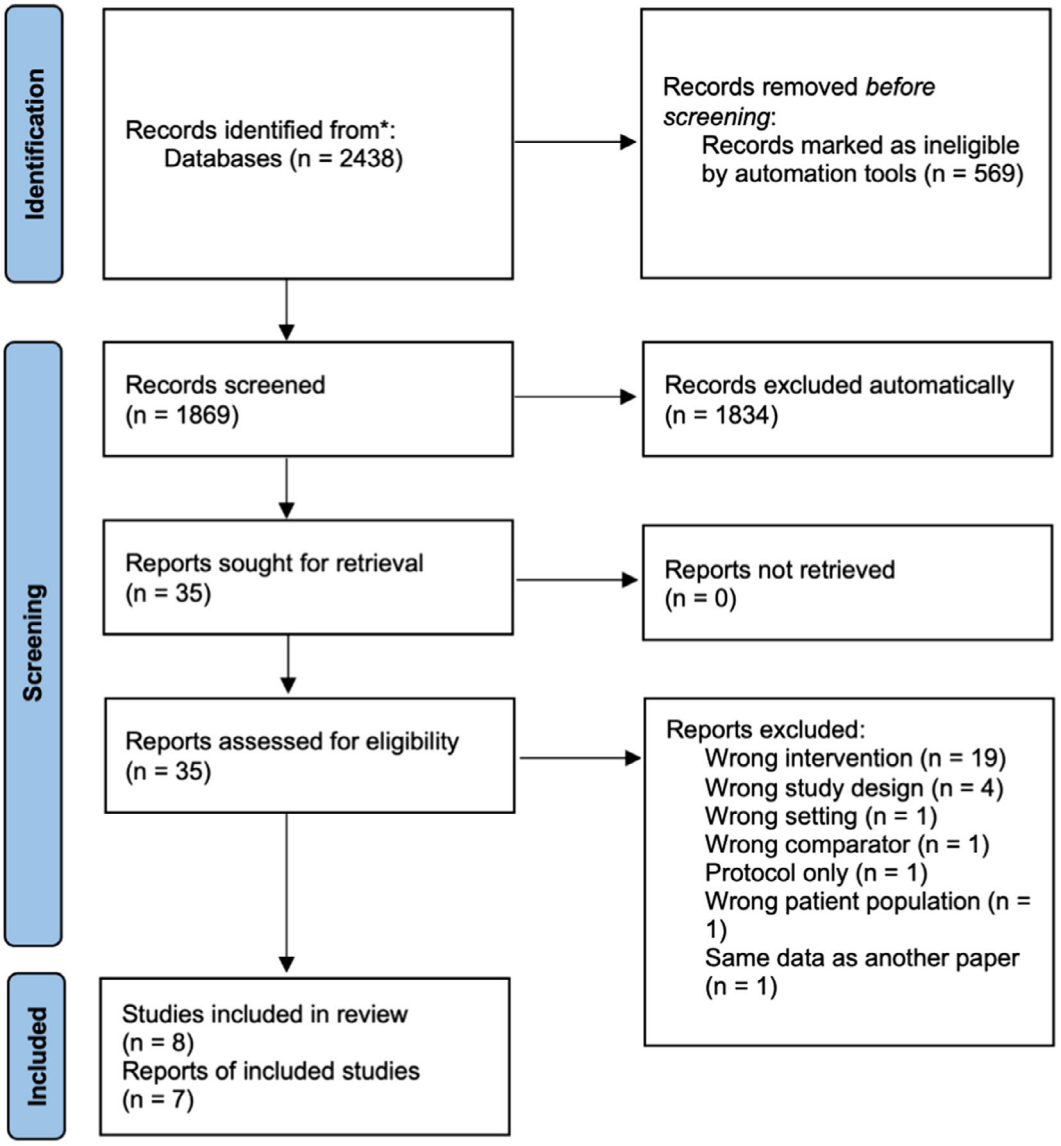
PRISMA flow chart

2438 papers were imported for screening. After duplicates were removed, 1869 papers were screened. 35 papers were screened at full‐text. Seven papers met the inclusion criteria and were included (Doty, Rudi, Pinna, Hanson, & Gewirtz, 2016; Dumas, Begle, French, & Pearl, 2010; Gross & Bettencourt, 2019; Heinrichs, 2006; Laxman, Higginbotham, & Bradford, 2019; Snow, Frey, & Kern, 2002; Stanger, Ryan, Fu, & Budney, 2011). Heinrichs (2006) reported a study comparing groups and individuals parenting programs with and without incentives. Because all four groups used different participants this was treated as 2 RCTs (one of groups and one of individuals) for the purposes of this review (Heinrichs, 2006). We labeled the comparison of the two individual arms (with and without incentives) ‘Heinrichs 1’ and the comparison of the two group arms (with and without incentives) ‘Heinrichs 2’, recombining data published in two papers (Heinrichs, 2006; Heinrichs & Jensen‐Doss, 2010) to give a fuller picture of two head‐to‐head RCTs, without double‐counting any data. Thus, overall we included eight unique cohorts each comparing one group with financial incentives with one group without. Reasons for exclusion are given in Appendix S2. Two papers (Doty et al., 2016; Laxman et al., 2019) did not present raw data relating to the impact of incentives on engagement so authors were contacted and data was obtained for both studies (Figure 1).

### Study characteristics

The seven included papers representing eight studies on unique cohorts were published between 2002 and 2019. Two were conducted in Germany and all others were conducted in the USA. Five of the eight studies used individual level randomization and three used quasi‐randomized designs assigning parents based on order of enrolment. Five studies set a threshold used for attendance at completing all modules and one study set a threshold at completing one module. Two studies did not include any binary threshold.

699 parents participated in control arms and 1862 participated in incentive arms. Mean control group size was 87 (range 19–291) and mean incentive group size was 233 (range 23–936).

### Participant demographics

Studies reported demographics inconsistently. Five reported the proportion of parents who were mothers (mean 62%, range 0%–93%). 5 studies reported the proportion of parents who were seeking to improve behavior of sons as opposed to daughters (mean 55%, range 53%–56%). The mean age of children was 4.9 (range 3.7–7.7) across five studies. The average age for parents was 32 years old (range 29–38) across six studies. The mean proportion of single parents was 37% (range = 8%–77%).

Six studies reported on the race or ethnicity of participants. The mean proportion of participants from minoritized ethnic or racial groups (predominantly African American or Hispanic/Latinx) was 39% (range 10%–97%). Only US studies reported race or ethnicity. Three studies reported percentage of participants who were immigrants. In Heinrichs' 2 studies (Heinrichs, 2006) 37% of participants were immigrants to Germany and in the Gross and Bettencourt (2019) study 28% of parents were immigrants to the USA. No studies had inclusion criteria based on children's diagnoses or severity of children's behavior problems. Appendix S3 summarizes the demographics of the populations of the included studies.

### Income, education and family composition

Income and educational level were reported inconsistently across studies. For example, Dumas et al. (2010) reported a mean annual household income of $25,850, standard deviation $13,136.35 (today: $38,000 with SD $19,200). Heinrichs (2006) reported 55% of participants across both studies had a household annual income below 18,000 Euros (today: $33,600), which was described as ‘low income’. Gross and Bettencourt (2019) found 95% of participants had a household income below $40,000 (today: $56,700). In contrast, Doty et al. (2016) studied military families and found only 26% of participants had a household income below $50,000 (today:$66,200).

Three studies reported participants' educational level. 23% of Heinrichs' (2006) participants had more than 10 years of education (Heinrichs, 2006). 50% of Doty et al.'s (2016) participants had a four year degree or more. 43% Laxman et al.'s (2019) participants had a bachelors or advanced degree.

Two studies reported rates of parental psychiatric disorder. In Stanger et al. (2011), all parents had a background of psychiatric disorder because it was an inclusion criterion. In Laxman et al. (2019), 8% of parents reported a diagnosis of a serious mental illness.

### Parenting program and incentive design

All studies used different parenting programs (ADAPT, STEP, Triple P, PACE, Incredible Years BASIC parent training program, Chicago Parenting Program, Fathering with Love and Logic, Home Run Dads, and 24/7 Dads) each lasting between 6 and 14 sessions.

Seven studies used incentives with a positive framing (incentive presented as extra money). Snow et al. (2002) used a negative framing (incentive matched to the price of the parenting program book which all participants bought upon enrolling and presented as reimbursement). Six studies used guaranteed incentives only. Stanger et al. (2011) used a lottery system only. Doty et al. (2016) combined guaranteed incentives with lottery incentives.

Table 1 shows that the expected value of incentives for a parent who attends all sessions varies from $16 to $270 (or $28 to $360 as updated for USD in 2024).

**Table 1:** Characteristics and results of included papers

Study	Incentive design	*n* ^e^ (study design)	Demographics^f^	Target population (child behavior/diagnosis inclusion/exclusion criteria)	Parenting program (sessions)	Maximum EV^g^ (adjusted^h^)	Odds ratio	Hedges' *g* (95%CI)
Doty et al. (2016)^d^	Parents received a guaranteed $15 incentive for attending each of 14 sessions. For each of 13 online assignments they had a roughly 25% chance of winning a $25 incentive.	74 vs. 288 (QES)	52% female parent 9.5% racial or minoritized ethnicity	Military families where at least one parent has deployed to Afghanistan or Iraq. (No criteria)	ADAPT (14 sessions)	$270^a^ ($358)	1.58	–
Dumas et al. (2010)	Parents received $3 for attending each of the first two sessions, $6 for the next two sessions, $10 for the next two sessions, and $15 for the final two sessions.	291 vs. 319 (Cluster RCT)	93% female parent 35% Black in control group, 61% Black in incentive group	Ethnically and socioeconomically diverse day care centers. (No criteria)	PACE (8 sessions)	$68 ($99)	1.15	0.05 (−0.54 to 0.21)
Gross and Bettencourt (2019)	Discount off childcare bill for attendance at each of 12 sessions. If total childcare costs <$5 then discount = costs; if total childcare costs >$5 then discount = $4 + 20% of total childcare costs.	397 vs. 395 (Cluster RCT)	89% female parent 55% Black, 42% of Latino	Childcare centers where 90% of parents are low income. (No criteria)	Chicago Parenting Program (12 sessions)	($107)^b^ ($152)	–	0.09 (−0.21 to 0.38)
Heinrichs (1) (2006)	10 Euros for each of 8 sessions plus 30 Euro perfect attendance bonus	168 vs. 182 (Cluster RCT)	72% at least one parent was an immigrant to Germany	Parents with children in preschools in ‘the socially most disadvantaged area of a mid‐sized city’. (No criteria)	Triple P (8 sessions)	110 Euros ($206)	1.48	0.41 (0.06 to 0.77)
Heinrichs (2) (2006)	15 Euros for each of 4 groups plus 30 Euro perfect attendance bonus	186 vs. 154 (Cluster RCT)	72% at least one parent was an immigrant to Germany	Parents with children in preschools in ‘the socially most disadvantaged area of a mid‐sized city’. (No criteria)	Triple P (4 sessions)	110 Euros ($206)	1.26	−0.16 (−0.54 to 0.21)
Laxman et al. (2019)^d^	$25 cards for attendance were offered either twice or on six occasions during the program.	104 vs. 571 vs. 365 (QES)	8.9% Latino, 4.6% other minoritized ethnicity, 1.9% multiple races	From the general public via ‘mailings, billboards, radio ads, partnerships with community organizations, the extension system, etc’. (No criteria)	Fathering with Love & Logic, Home Run Dads, 24/7 Dads (5 sessions)	$50 or $150 ($62–$187)	1.09	–
Snow et al. (2002)	Participants received $16 for attending all 10 sessions.	37 vs. 42 (RCT)	11% Black, 3% Hispanic, 3% Asian American, 1% Native American	Recruited by school counselors through voluntary sign‐up sheets. (No criteria)	STEP (10 sessions)	$16 ($28)	0.92	–
Stanger et al. (2011)	Lottery with EV of $2.44 including 1/250 change of $100. 2 pulls for each session, 1 pull per phone call, plus 5 pulls if more calls taken than previous week.	19 vs. 28 (RCT)	29% African American, 2% multiethnic	Referred by substance abuse agencies, courts, or self‐referred. (No criteria)	Incredible Years (12 sessions)	($252)^c^ ($359)	‐	−0.25 (−0.82 to 0.33)

EV, expected value; QES, quasi‐experimental study; RCT, Randomized Controlled Trial; STEP, Systematic Training for Effective Parenting.

^a^ The EV of homework assignments varied based on the size of the group.

^b^ EV depends on childcare costs. Average weekly cost was $8.92 so average EV for perfect attendance is $107.04.

^c^ Maximum income scored by beating previous week's attendance not perfect engagement. Actual mean total score of $252.

^d^ Quasi‐randomized design.

^e^ Order of number indicates control then intervention, except in Laxman et al. where it is control, then intervention 1, then intervention 2.

^f^ Demographics described in keeping with descriptions in the original research paper.

^g^ Maximum EV converted to USD and updated for June 2024.

^h^ Incentive EV if perfect attendance and completion of study requirements.

### Risk of bias within studies

Five randomized controlled trials scored between 6 and 8 out of 11 on the JBI RCT assessment tool (JBI, 2020a; Tufanaru, Munn, Aromataris, Campbell, & Hopp, 2020), indicating minimal bias within studies. Lack of blinding, nonsimilar groups, and nonconcealed group allocation were excluded for the following reasons: A. Blinding to the incentive, if it were possible, would undermine the ecological validity of the study. B. Because we are studying whether participants join studies where they are going to be offered incentives, nonconcealed group allocation is an important way we could answer our research questions. C. The existence of nonsimilar groups is an outcome of interest with respect to research question 2. These features do not increase risk of bias because our analysis begins upstream of this point, at recruitment. Aside from these specific exclusions, the JBI risk of bias tools were considered appropriate for evaluating these studies. The three quasi‐experimental studies scored 6 or 7 out of 9 on the JBI quasi‐experimental assessment tool (JBI, 2020b; Tufanaru et al., 2020). Points were lost because measurements were not made pre‐ and post‐ exposure (which is irrelevant in this context) and also because demographics were not reported separately for control and intervention groups. (See Appendixes S4.1 and S4.2.)

#### Research Question 1: Do financial incentives for engagement in parenting programs affect parental engagement

##### Connection

**Figure 2:**
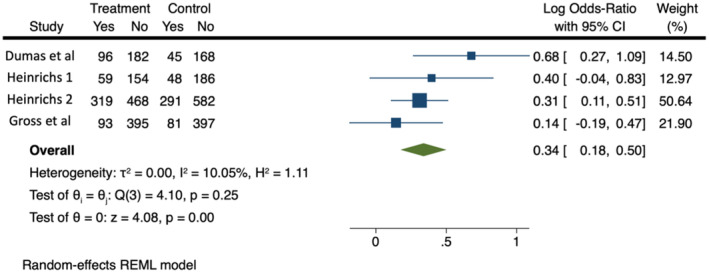
Meta‐analysis 1: Proportions of invited parents who agreed to participate. CI, confidence interval; REML, restricted maximum likelihood

Four studies informed parents which arm they would be in prior to participation and reported how many parents were invited to each group. Figure 2 shows that in all four studies reporting this measure, a greater proportion of invited people agreed to participate in the incentive group. The effect was most potent in Heinrichs' one‐to‐one (Heinrichs, 2006) study but persisted in all four studies. Dumas et al. (2010) had much higher rates of connection with invited participants in both arms compared with the other studies. In meta‐analysis 1, invited people were more likely to participate when they knew they were entering the incentive program (OR = 1.40, 95% CI 1.20–1.65, Log Odds Ratio = 0.34, 95% CI 0.18–0.50). There was low heterogeneity (*I* ^2^ = 10.05, *Q* = 4.1 *p* = .25). The 95% prediction interval for the OR was 0.91–2.16.

##### Attendance

All studies included a measure of attendance. This was either a binary measure of the number of parents who attended a given threshold of sessions or a mean number of sessions attended. Appendix S5 includes the raw data and the log odds ratios and/or hedges' *g* for each of the eight studies.

**Figure 3:**
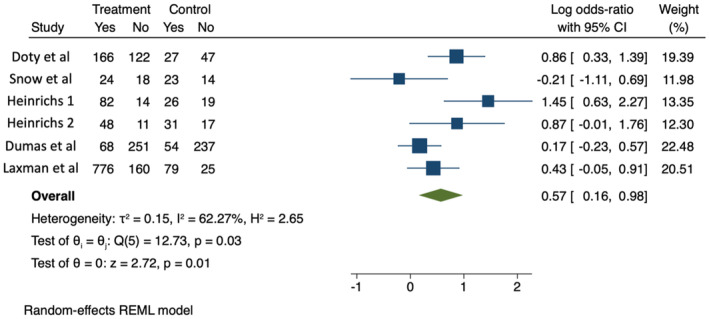
Meta‐analysis 2: Proportions of participating parents who reached attendance threshold

In meta‐analysis 2 (see Figure 3), odds ratios comparing the number of participants completing a certain threshold of modules varied from 0.81 (the only negative result) to 4.26. Meta‐analysis generated an odds ratio of 1.76 (95% CI: 1.17–2.66, log odds ratio 0.57, 95% CI: 0.16–0.98). *I* ^2^ was 62.27%. (*Q*(5) = 12.73, *p* = .03), indicating that incentives were associated with increased odds of participation past the threshold point. The 95% prediction interval for the OR was 0.52–6.05.

Five studies reported the mean number of modules completed with and without incentives. Three larger studies found people in the incentive group completed more modules than controls. Two smaller studies (Heinrichs' group comparison (2010) and Snow et al.'s study (2002)) found lower mean module completion among treatment groups compared with controls. Meta‐analysis 3 (see Appendix S6) found Hedges' *g* was 0.06 (95% CI: −0.08 to 0.21). The *I* ^2^ was 17.8% (*Q*(4) = 6.3, *p* = .18). The 95% prediction interval for Hedges' *g* was −0.27 to 0.40.

There was insufficient evidence regarding the participate phase enact phase for any conclusions to be drawn. As well as incentivizing group attendance, Stanger et al. (2011) also incentivized check in phone calls and found 41% of participants in the incentive group made check in phone calls compared with 21% of participants in the control group. The authors found no difference in the number of homework assignments completed. No measures of parental behavior change were reported.

##### Combining connection and attendance

Three studies reported both connection (the proportion invited to each group who agreed to participate) and attendance (the proportion of participants in each group who completed the program). In all three, incentives had a positive effect on both connection and in two incentives had a positive effect on attendance. When connection and attendance were combined the overall effect of incentives was positive in all three studies. In meta‐analysis 4 (see Appendix S7.2) the odds ratio of the combined effect was 2.51 (95% CI: 1.42–4.48, log odds ratio 0.92, 95% CI: 0.35–1.50). *I* ^2^ was 78.25%. The 95% prediction interval for the OR was −5.91 to 2344.90. The significant result of meta‐analysis 4 (see Appendixes S7.1 and S7.2) is consistent with meta‐analyses 1, 2, and 3 where the point estimates for the effect of financial incentives on participation and engagement were positive.

No studies compared participation during groups between incentive and control groups.

#### Research Question 2: Do financial incentives for engagement in parenting programs lead to increased engagement among groups experiencing structural inequities, including racism, discrimination, and poverty?

Several studies reported differences regarding connection by race, income and family composition, but none reported differences in attend, participate or enact. Most studies indicated the demographics of the whole sample (described above), but only 3 give any information about demographic differences between incentive and control groups.

Dumas et al. (2010) reported that a higher percentage of Black or African American people joined the incentive group than the control group (61% of the incentive group vs 35% of the control group). Given that prospective participants received an invitation to either the incentive or control group at random, this indicates that there was a relatively higher amount of interest in the incentive group among Black or African American people compared to the control group. Gross and Bettencourt (2019) found different ethnic and racial differences; there was a slightly lower proportion of Black or African American people in the incentive (53%) than the control (58%) group, but a larger proportion of Hispanic/Latinx participants in the incentive group than control group (45% vs. 38%). Dumas et al. (2010) also evaluated the proportion of participants who were immigrants to the USA and a greater proportion of immigrants joined the intervention group than the control group (36% vs. 20%). Laxman et al. (2019) found fewer white people in the incentive group than the control group (84.3% vs. 86.5%).

Dumas et al. (2010) found household income was lower in the incentive group compared with those who joined the control group ($22,000 vs. $29,000, today: $31,500 vs. $42,000). Gross and Bettencourt (2019) found the median annual household income in both groups was between $10,000 and $14,999 (today: $14,200 and $21,300) but hourly income was lower among parents who agreed to join the incentive group ($10.80 vs. $12.08, today: $15.41 vs. $17.23). Laxman et al. (2019) also found a higher annual income in the control group than the incentive group ($39,600 vs. $34,600, today: $49,400 vs. $43,100).

Dumas et al. (2010) found more single parents joined the incentive group (64% vs. 46%) while Laxman et al. (2019) found a slightly larger proportion of single parents in the incentive group (5.5% vs. 8.8%). No differences were found regarding parent age and gender, nor child age.

#### Research Question 3: Have outcomes of financial incentives differed based on their design; for example, the size of the incentives, whether they are certain or uncertain, and whether they use cash or vouchers?

One study compared different incentive designs. Laxman et al. (2019) found lower disengagement (defined as leaving the program and not returning) among those offered 6 gift cards with a total value of $150 (today: $187, 13% disengaged) compared with those offered two gift cards with a total value $50 (today: $62, 20% disengaged). Heinrichs' two studies (Heinrichs, 2006) allow comparison between attendance at 8 group sessions and at 4 one to one sessions. When attendance at the more frequent sessions was incentivized, attendance was affected more on both measures, compared with when attendance at the less frequent session was incentivized.

Two of the remaining studies observed that the incentive group had lower engagement than the control group (Snow et al., 2002; Stanger et al., 2011). Both had values of incentive below the hourly US federal minimum wage. Snow et al. offered a guaranteed $16 (today: $28) for attending all sessions and Stranger et al offered lottery tickets with an expected value of $4.88 (today: $6.95) per session attended, both falling below the US federal minimum wage of $7.25 per hour (State Minimum Wage Laws, 2024). Stranger et al offered more tickets for check in phone calls and the incentive group took more calls than the control group. The 3 studies with the strongest positive effect of the incentive on engagement (Doty et al. (2016) and both studies by Heinrichs (2006)) all offered all parents guaranteed incentives worth today $20 or more for each session.

The results of studies with quasi‐experimental designs did not differ from the results of experimental studies. Doty et al. (2016) found a strong positive effect of incentives on engagement, consistent with the findings from Heinrichs and Jensen‐Doss (2010), Laxman et al., found a small positive effect of incentives on engagement consistent with the findings from Dumas et al. Lastly, Snow et al. found a negative result of incentives on engagement like Stranger et al.

#### Research Question 4: Do financial incentives for engagement at parenting programs lead to improved child behavior?

Three studies (Heinrichs, 2006; Stanger et al., 2011) reported the child behavior outcomes in each group. Stranger et al conducted intention to treat analysis and reported pre and post measures of child behavior problems (measured with CBCL) in each group finding that externalizing behavior problems improved more in the incentive group, but did not find any difference in internalizing behaviors (Stanger et al., 2011). No significant differences in parenting style were identified. A further paper (Heinrichs & Jensen‐Doss, 2010) reported father‐ and mother‐rated measures of child behavior problems (measured with the Strengths and Difficulties Questionnaire (SDQ)) as well as parenting scores from Heinrichs' studies, finding that father‐rated behavior problems were less improved in the incentive group but there was no difference in the mother‐rated scores.

### Risk of bias across studies

Funnel plots were generated for meta‐analyses 2 and 3 as they included the largest number of studies (Appendix S9.1 and S9.2). Visual evaluation of funnel plots gave no indication of bias across studies but should be interpreted with caution due to small numbers. It would not be informative to present funnel plots for small meta‐analyses of 3 or 4 studies.

### Summary of findings

**Table 2:** Summary of findings

Finding	Heading	Claim	Measure (95% CI)	Number of studies	GRADE^a^
1	Connection and Attendance	People invited to incentive group are more likely to reach threshold number of sessions.	2.51 (95% CI: 1.42–4.48)	3 or 8^b^	High certainty
2	Connection	More parents connect with parenting programs when they know there will be incentives for participation.	Odds ratio 1.40, 1.22–1.65	4	Moderate certainty
3	Attendance	Incentives increase how many parents reach threshold number of sessions.	Odds ratio 1.76 (1.17–2.66) Hedges' *g* 0.06 (−0.08 to 0.21)	6	Moderate certainty
4	Demographic differences (income)	Incentives increase connection among people with lower incomes.	n/a	3	Moderate certainty
5	Demographic differences (race, ethnicity, and immigration status)	Incentives increase connection among ethnically and racially minoritized groups as well as immigrant populations.	n/a	3	Low certainty
6	Enacting	Evidence suggests no difference in behavior change between incentive and control groups.	n/a	3	Low certainty
7^c^	Differences between incentive designs	Guaranteed incentives for each attendance valued over $10 are most effective at increasing engagement.	n/a	8	Low certainty

^a^ Full GRADE table provided in Appendix S10.

^b^ Three studies in the main meta‐analysis, but 8 studies across meta‐analyses 3 and 4 also provided supporting evidence.

^c^ The protocol specified that a summary of findings table would include participation but there was no finding regarding participation so it has been omitted.

## Discussion

### Summary of results

This preregistered systematic review and meta‐analysis included eight studies comparing engagement with parenting programs in groups with and without financial incentives. Beginning from the connect phase, parents invited to incentive arms were more likely to reach the threshold of sessions than parents invited to control arms (odds ratio 2.51 95% CI 1.42–4.48). The idea that parents invited to the incentive arm were more likely to complete sessions than those invited to nonincentive arms was the only finding drawn with high certainty. With regard to the attend phase, parents were more likely to participate when they knew they were joining the incentive program (odds ratio 1.40, 95% CI 1.20–1.65). Parents in the incentive group were more likely than parents in the control group to reach a completion threshold of sessions (odds ratio 1.76, 95% CI: 1.17–2.66) although the increase in the number of sessions attended was not statistically significant (Hedges' *g* = 0.06, 95% CI: −0.08–0.21). There was insufficient evidence to comment on the participate phase or enact phase.

Six studies found that financial incentives were associated with better engagement among people from minoritized ethnic and racial groups and among immigrant populations than other groups, and three studies found lower incomes among participants in financial incentive arms (vs control arm), possibly because parents with lower incomes were more likely to consent to participation if they were allocated to arms with financial incentives. Although the many differing elements of incentive design made it difficult to ascertain any relationship between incentive designs and effects on engagement, our findings suggested that guaranteed incentives worth $10 or more for each attendance were associated with better engagement compared to less regular and smaller incentives. With regard to whether financial incentives for engagement lead to improved child behavior among children whose parents participated, existing evidence was limited but suggested outcomes were no worse among those whose parents were attended sessions with incentives and those who attended without incentives.

### Comparison with the literature

#### Comparison with other approaches to increasing engagement

The increase in engagement caused by financial incentives appears to be more robust than the increase prompted by motivational interviewing or changing delivery to digital to reduce barriers. Prinz and Miller studied 147 families and found those randomized to receive additional therapist support attended 71% of sessions, compared with 53% without (Ingoldsby, 2010). Similarly two RCTs of Strategic Structural‐Systems Engagement, a brief intervention building on structural family therapy, found increased engagement (77% vs. 25%, 72% vs. 42%) (Ingoldsby, 2010). While both interventions were large and indicated clinically significant effects, the additional cost of therapist time is likely to restrict access to these approaches within resource constrained settings, certainly compared to $10–$20 financial incentives. Online programs also have the potential to ease access although engagement is low (Dadds et al., 2019). The largest study found 7% of referred parents completed the program whereas comparable figures from the present study ranged from 14% to 45%.

#### Range of financial incentives across healthcare which have changed behavior

We found that financial incentives doubled the odds of invited participants completing the program. Broadly similar findings were reported from a systematic review of studies where financial incentives were used to improve engagement with HIV testing (RR 2.42, 95% CI 1.06–5.54) (Krishnamoorthy, Rehman, & Sakthivel, 2021). A systematic review found that financial incentives were the most effective intervention to increase flu vaccine uptake among people with chronic disease (RR 2.79, 95% CI: 1.18–6.62) (Sanftenberg, Brombacher, Schelling, Klug, & Gensichen, 2019). Elsewhere in mental health, there is consistent evidence of increased antipsychotic depot injection acceptance among patients with psychosis (Hodson, 2022; Noordraven et al., 2017; Priebe et al., 2013).

#### People with less access to resources appear more engaged by incentives than high income people

In the absence of incentives, program completion is lower among low‐income families. Our findings suggest that, compared with high income parents, low‐income parents' behavior is more influenced by financial incentives (moderate certainty). Incentives could therefore potentially target programs at parents with low income. Among parents with higher incomes incentives may reduce motivation to complete programs, despite increasing program completion among parents with lower incomes. Further study of this phenomenon is required and the ethical implications merit consideration.

### Strengths and limitations

To our knowledge, this is the first systematic review to appraise the effect of financial incentives on parenting program engagement. By evaluating the change from invitation to participate the review has relevance to the real‐world challenge of ensuring parents who are invited to parenting programs engage. The review's findings are in keeping with the evidence from behavioral economics and provide the meta‐analytic evidence to support the policy of using incentives in parenting programs.

Although data from a reasonably large number of participants were included, only eight studies were included and their designs were heterogeneous. This combination of a heterogeneity and a small number of studies may explain why the number of modules attended trended in the same direction as the proportion of participants reaching a threshold, but did not reach significance. Included studies were disproportionately from settings in the United States. The included studies selected participants pragmatically based on routine pathways and did not restrict inclusion to parents whose children had received a formal diagnosis of a disruptive behavioral disorder. Although parenting skills research increasingly focuses on digital innovations only one included study had an online component (Day et al., 2021). Finally, no formal cost‐effectiveness analysis was conducted, restricting our ability to comment on the cost of each additional parent‐session attended.

### Implications for research, policy and practice

Replications of these studies should be conducted with the parents of children who have diagnoses of conduct disorder or ADHD. Future researchers should consider exploring how incentives are explained to parents (e.g., payments, travel expenses, signs of gratitude, or rewards) (Hodson, Majid, Vlaev, & Singh, 2023). Parents' interpretations of incentives should also be investigated regarding whether they signal that the program is useless or unpleasant (Hodson, Majid, Vlaev, & Singh, 2023). We also suggest optimizing incentive design by testing different magnitudes, frequencies, lottery arrangements, and loss/gain framing in randomized trials (Vlaev et al., 2019).

We recommend parenting programs consider financial incentives to increase program engagement. Policymakers will have to consider who pays for incentives and future research should consider cost–benefit analysis of parenting programs with and without incentives. Future studies should evaluate the effect of financial incentives on the participation and enact phases, as well as investigating whether there is any relationship between financial incentives and improvement in child behavior problems and the ethics of financial incentive use in populations from minoritized and marginalized backgrounds.

## Conclusions

This systematic review concluded with high certainty that incentives increase parenting program engagement among invited parents who have not yet attended and those who have started attending programs, especially with respect to parents from low‐income backgrounds and minoritized ethnic or racial groups. Incentives should be considered an effective potential tool for increasing engagement but further research is needed to establish acceptability. By increasing engagement among families currently missing out on parenting programs, financial incentives can contribute to mitigating the adverse outcomes of disruptive behavior disorders and improving the lives of affected children and their parents.

## Ethical information

Ethical approval is not applicable to this article as no new data were created or analyzed in this systematic review study.

## Supporting information


**Appendix S1.** Search strategies (as per protocol).
**Appendix S2.** List of papers excluded at full paper review with reasons, generated from Covidence.
**Appendix S3.** Population demographics of included studies.
**Appendix S4.1.** Risk of bias in quasi‐experimental studies.
**Appendix S4.2.** Risk of bias in randomized controlled trials.
**Appendix S5.** Parenting program attendance and financial incentives.
**Appendix S6.** Meta‐analysis 3: Mean module completion rates among participants.
**Appendix S7.1.** Relationship between financial incentives and proportion of participants reaching the completion threshold.
**Appendix S7.2.** Meta‐analysis 4: Proportions of invited parents who reached attendance threshold.
**Appendix S8.** How data from each included study fits the CAPE framework.
**Appendix S9.1.** Funnel plots showing the relationship between standard error and engagement outcomes.
**Appendix S9.2.** Funnel plots showing the relationship between standard error and engagement outcomes.

**Appendix S10.** Full GRADE table.

## Data Availability

No additional data available.
