# Supplementary material for: Review: Systematic review and meta‐analysis – financial incentives increase engagement with parenting programs for disruptive behavior problems
Source: Child Adolesc Ment Health. 2024 Dec 21;30(1):53–65. doi: 10.1111/camh.12746 (PMC11754718; doi:10.1111/camh.12746)
Supplement: Supplementary file 1 — Appendix S1. Search strategies (as per protocol). Appendix S2. List of papers excluded at full paper review with reasons, generated from Covidence. Appendix S3. Population demographics of included studies. Appendix S4.1. Risk of bias in quasi‐experimental studies. Appendix S4.2. Risk of bias in randomized controlled trials. Appendix S5. Parenting program attendance and financial incentives. Appendix S6. Meta‐analysis 3: Mean module completion rates among participants. Appendix S7.1. Relationship between financial incentives and proportion of participants reaching the completion threshold. Appendix S7.2. Meta‐analysis 4: Proportions of invited parents who reached attendance threshold. Appendix S8. How data from each included study fits the CAPE framework. Appendix S9.1. Funnel plots showing the relationship between standard error and engagement outcomes. Appendix S9.2. Funnel plots showing the relationship between standard error and engagement outcomes. [file CAMH-30-53-s001.docx]

**Appendixes**

***Appendix 1: Search strategies (as per protocol)***

*Pubmed*

("Economics, Behavioral"[Mesh] OR "Behavioral economics" OR "Behavioural economics" OR "Financial incentives" OR "Financial incentive" OR "Financial interventions" OR "Financial health incentives" OR "Payment incentive" OR "lottery-based incentive " OR "lottery-based incentives" OR "direct incentive" OR "direct incentives" OR "unconditional incentives" OR "Cash incentive" OR "Cash incentives" OR "Conditional cash transfer*" OR "monetary incentive*"[All Fields] OR "Incentive scheme*"[All Fields] OR "Incentive strateg*"[All Fields] OR "direct benefit transfer" OR "Economic incentives" OR "Incentive program*" OR "reimbursement, incentive"[Mesh]) AND

("Parenting"[All Fields] OR "parent*"[ti] OR "family"[ti] OR "parents/education"[Mesh] OR "parent education"[All Fields] OR mother*[All Fields] OR father*[All Fields] OR "family relations"[Mesh] OR "parent–child relationship*" OR "parenting program*" OR "parental engagement" OR "parenting skills" OR "parental skills" OR "parent-child interaction*" OR "parent participation" OR "parent training" OR "parenting effectiveness" OR "parenting strategies" OR "family engagement" OR "family engaged" OR "parent engagement" OR "parental engagement" OR "parent engaged" OR "parenting intervention*" OR "family intervention*" OR "family based" OR "family centered")

*CINAHL*

( "Behavioral economics" OR "Behavioural economics" OR "Financial incentives" OR "Financial incentive" OR "Financial interventions" OR "Financial health incentives" OR "Payment incentive" OR "lottery-based incentive " OR "lottery-based incentives" OR "direct incentive" OR "direct incentives" OR "unconditional incentives" OR "Cash incentive" OR "Cash incentives" OR "Conditional cash transfer*" OR "monetary incentive*" OR "Incentive scheme*” OR "Incentive strateg*" OR "direct benefit transfer" OR "Economic incentives" OR "Incentive program*" OR MH "reimbursement, incentive") AND

(MH "Parenting" OR TI "parent*" OR TI "family" OR MH "parents/ED" OR "parent education" OR mother* OR father* OR MH "family relations+” OR "parent–child relationship*" OR "parenting program*" OR "parental engagement" OR "parenting skills" OR "parental skills" OR "parent-child interaction*" OR "parent participation" OR "parent training" OR "parenting effectiveness" OR "parenting strategies" OR "family engagement" OR "family engaged" OR "parent engagement" OR "parental engagement" OR "parent engaged" OR "parenting intervention*" OR "family intervention*" OR "family based" OR "family centered") Filters: English language, Academic Journals

*PsycINFO*

( "Behavioral economics" OR "Behavioural economics" OR "Financial incentives" OR "Financial incentive" OR "Financial interventions" OR "Financial health incentives" OR "Payment incentive" OR "lottery-based incentive " OR "lottery-based incentives" OR "direct incentive" OR "direct incentives" OR "unconditional incentives" OR "Cash incentive" OR "Cash incentives" OR "Conditional cash transfer*" OR "monetary incentive*" OR "Incentive scheme*” OR "Incentive strateg*" OR "direct benefit transfer" OR "Economic incentives" OR "Incentive program*" OR "reimbursement, incentive") AND

("Parenting" OR "parent*" OR "family" OR "parents/ED" OR "parent education" OR mother* OR father* OR "family relations” OR "parent–child relationship*" OR "parenting program*" OR "parental engagement" OR "parenting skills" OR "parental skills" OR "parent-child interaction*" OR "parent participation" OR "parent training" OR "parenting effectiveness" OR "parenting strategies" OR "family engagement" OR "family engaged" OR "parent engagement" OR "parental engagement" OR "parent engaged" OR "parenting intervention*" OR "family intervention*" OR "family based" OR "family centered")

*Sociological Abstracts*

(SU( "Behavioral economics" OR "Behavioural economics" OR "Financial incentives" OR "Financial incentive" OR "Financial interventions" OR "Financial health incentives" OR "Payment incentive" OR "lottery-based incentive " OR "lottery-based incentives" OR "direct incentive" OR "direct incentives" OR "unconditional incentives" OR "Cash incentive" OR "Cash incentives" OR "Conditional cash transfer*" OR "monetary incentive" OR "monetary incentives" OR "incentive scheme" OR "incentive schemes" OR "Incentive strateg*" OR "direct benefit transfer" OR "Economic incentives" OR "incentive program" OR "incentive programs" OR "reimbursement, incentive") OR TIAB( "Behavioral economics" OR "Behavioural economics" OR "Financial incentives" OR "Financial incentive" OR "Financial interventions" OR "Financial health incentives" OR "Payment incentive" OR "lottery-based incentive " OR "lottery-based incentives" OR "direct incentive" OR "direct incentives" OR "unconditional incentives" OR "Cash incentive" OR "Cash incentives" OR "Conditional cash transfer*" OR "monetary incentive" OR "monetary incentives" OR "incentive scheme" OR "incentive schemes" OR "Incentive strateg*" OR "direct benefit transfer" OR "Economic incentives" OR "incentive program" OR "incentive programs" OR "reimbursement, incentive")) AND

("Parenting" OR "parent*" OR "family" OR "parents" OR "parent education" OR mother* OR father* OR "family relations” OR "parent–child relationship*" OR "parenting program" OR "parenting programs" OR "parental engagement" OR "parenting skills" OR "parental skills" OR "parent-child interaction*" OR "parent participation" OR "parent training" OR "parenting effectiveness" OR "parenting strategies" OR "family engagement" OR "family engaged" OR "parent engagement" OR "parental engagement" OR "parent engaged" OR "parenting intervention*" OR "family intervention" OR "family based" OR "family centered")

*Cochrane Trials*

( "Behavioral economics" OR "Behavioural economics" OR "Financial incentives" OR "Financial incentive" OR "Financial interventions" OR "Financial health incentives" OR "Payment incentive" OR "lottery-based incentive " OR "lottery-based incentives" OR "direct incentive" OR "direct incentives" OR "unconditional incentives" OR "Cash incentive" OR "Cash incentives" OR "Conditional cash transfer*" OR "monetary incentive" OR "monetary incentives" OR "incentive scheme" OR "incentive schemes" OR "Incentive strateg*" OR "direct benefit transfer" OR "Economic incentives" OR "incentive program" OR "incentive programs" OR "reimbursement, incentive" OR "Behavioral economics" OR "Behavioural economics" OR "Financial incentives" OR "Financial incentive" OR "Financial interventions" OR "Financial health incentives" OR "Payment incentive" OR "lottery-based incentive " OR "lottery-based incentives" OR "direct incentive" OR "direct incentives" OR "unconditional incentives" OR "Cash incentive" OR "Cash incentives" OR "Conditional cash transfer*" OR "monetary incentive" OR "monetary incentives" OR "incentive scheme" OR "incentive schemes" OR "Incentive strateg*" OR "direct benefit transfer" OR "Economic incentives" OR "incentive program" OR "incentive programs" OR "reimbursement, incentive") AND ("Parenting" OR "parent*" OR "family" OR "parents" OR "parent education" OR mother* OR father* OR "family relations” OR "parent–child relationship*" OR "parenting program" OR "parenting programs" OR "parental engagement" OR "parenting skills" OR "parental skills" OR "parent-child interaction*" OR "parent participation" OR "parent training" OR "parenting effectiveness" OR "parenting strategies" OR "family engagement" OR "family engaged" OR "parent engagement" OR "parental engagement" OR "parent engaged" OR "parenting intervention*" OR "family intervention" OR "family based" OR "family centered")

***Appendix 2: List of papers excluded at full paper review with reasons, generated from Covidence.***

**#751 - Fernald 2008**

Fernald, L. C. H., Gertler, P. J., & Neufeld, L. M. (2008). Role of cash in conditional cash transfer programmes for child health, growth, and development: An analysis of Mexico's Oportunidades. The Lancet, 371(9615), 828-837. https://doi.org/10.1016/S0140-6736(08)60382-7

**Wrong intervention**

**#907 - Lachman 2021**

Lachman, J. M., Alampay, L. P., Jocson, R. M., Alinea, C., Madrid, B., Ward, C., Hutchings, J., Mamauag, B. L., Garilao, M. A. V. F. V., & Gardner, F. (2021). Effectiveness of a parenting programme to reduce violence in a cash transfer system in the Philippines: RCT with follow-up. The Lancet Regional Health - Western Pacific, 17, 100279.

DOI: 10.1016/j.lanwpc.2021.100279 · Ref ID: 34734199

**Wrong intervention**

Kamon, J., Budney, A., & Stanger, C. (2005). A contingency management intervention for adolescent marijuana abuse and conduct problems. Journal of the American Academy of Child & Adolescent Psychiatry, 44(6), 513-521. https://doi.org/10.1097/01.chi.0000159949.82759.64

**Wrong study design**

**#129 - Lopez-Arana 2016**

Lopez-Arana, S., Avendano, M., van Lenthe, F. J., & Burdorf, A. (2016). The impact of a conditional cash transfer programme on determinants of child health: Evidence from Colombia. Public Health Nutrition, 19(14), 2629-2642. https://doi.org/10.1017/S1368980016000240

**Wrong intervention**

**#4 - Hill 2021**

Hill, Z., Spiegel, M., Gennetian, L., Hamer, K.-A., Brotman, L., & Dawson-McClure, S. (2021). Behavioral economics and parent participation in an evidence-based parenting program at scale. Prevention Science, 22(7), 891-902. https://doi.org/10.1007/s11121-021-01249-0

**Wrong intervention**

#1897 - NCT01736995 2012

Oregon Research Institute. (2012). Family and Adolescent Motivational Incentives for Leveraging Youth (FAMILY) [Clinical trial]. ClinicalTrials.gov. https://clinicaltrials.gov/ct2/show/NCT01736995

**protocol only**

**#1257 - Serván-Mori 2022**

Serván-Mori, E., Pineda-Antúnez, C., Bravo-Ruiz, M. L., Molina, M., Ramírez-Baca, M. I., García-Martínez, A., Quezada-Sánchez, A. D., & Orozco-Núñez, E. (2022). A behavioral economics analysis of the participation in early childhood development social programs promoted by civil societies in Mexico. PLoS One, 17(3), e0265389. https://doi.org/10.1371/journal.pone.0265389

**Wrong study design**

**#563 - Boller 2015**

Boller, K., Paulsell, D., Grosso, P. D., Blair, R., Lundquist, E., Kassow, D. Z., Kim, R., & Raikes, A. (2015). Impacts of a child care quality rating and improvement system on child care quality. Early Childhood Research Quarterly, 30(Part B), 306-315. https://doi.org/10.1016/j.ecresq.2014.10.001

**Wrong intervention**

**#26 - Kagawa 2017**

Kagawa, R. M. C., Deardorff, J., García-Guerra, A., Knauer, H. A., Schnaas, L., Neufeld, L. M., & Fernald, L. C. H. (2017). Effects of a parenting program among women who began childbearing as adolescents and young adults. Journal of Adolescent Health, 61(5), 634-641. https://doi.org/10.1016/j.jadohealth.2017.05.023

**Wrong intervention**

**#387 - Gennetian 2019**

Gennetian, L. A., Marti, M., Kennedy, J. L., Kim, J. H., & Duch, H. (2019). Supporting parent engagement in a school readiness program: Experimental evidence applying insights from behavioral economics. Journal of Applied Developmental Psychology, 62, 1-10. https://doi.org/10.1016/j.appdev.2018.12.006

**Wrong intervention**

**#37 - Gross 2019**

Gross, D., & Bettencourt, A. F. (2019). Financial incentives for promoting participation in a school-based parenting program in low-income communities. Prevention Science, 20(4), 585-597. https://doi.org/10.1007/s11121-019-0977-y

**Wrong study design**

**#373 - Huang 2019**

Huang, J., Beverly, S. G., Kim, Y., Clancy, M. M., & Sherraden, M. (2019). Exploring a model for integrating child development accounts with social services for vulnerable families. Journal of Consumer Affairs, 53(3), 770-795. https://doi.org/10.1111/joca.12239

**Wrong intervention**

#221 - Fernald 2009

Fernald, L. C. H., Gertler, P. J., & Neufeld, L. M. (2009). 10-year effect of Oportunidades, Mexico's conditional cash transfer programme, on child growth, cognition, language, and behaviour: A longitudinal follow-up study. The Lancet, 374(9706), 1997-2005. https://doi.org/10.1016/S0140-6736(09)61676-7

**Wrong intervention**

**#365 - Piper 2020**

Piper, M. E., Brown, D. C., Hendershot, T. P., & Swan, G. E. (2020). PhenX: Host: Social/cognitive measures for tobacco regulatory research. Tobacco Control: An International Journal, 29(Suppl 1), s5-s12. https://doi.org/10.1136/tobaccocontrol-2018-054467

**irrelevant**

**#680 - Fernald 2011**

Fernald, L. C. H., & Hidrobo, M. (2011). Effect of Ecuador's cash transfer program (Bono de Desarrollo Humano) on child development in infants and toddlers: A randomized effectiveness trial. Social Science & Medicine, 72(9), 1437-1446. https://doi.org/10.1016/j.socscimed.2011.03.005

**Wrong intervention**

**#369 - Litwin 2019**

Litwin, A., Perova, E., & Reynolds, S. A. (2019). A conditional cash transfer and women's empowerment: Does Bolsa Familia influence intimate partner violence: Corrigendum. Social Science & Medicine, 241, 112579. https://doi.org/10.1016/j.socscimed.2019.112579

**Wrong patient population**

**#1464 - Gitter 2009**

Gitter, S. R., & Barham, B. L. (2009). Conditional cash transfers, shocks, and school enrolment in Nicaragua. The Journal of Development Studies, 45(10), 1747-1767. https://doi.org/10.1080/00220380902935857

**Wrong intervention**

**#396 - Reininger 2019**
Reininger, T., Villalobos, C., & Wyman, I. (2019). CCTs and conditionalities: An exploratory analysis of not meeting conditional cash transfer conditionalities in Chile’s Families Programme. Journal of Poverty and Social Justice, 27(1), 95-114. https://doi.org/10.1332/175982718X15375193954389

Wrong intervention

**#232 - Axford 2012**
Axford, N., Lehtonen, M., Kaoukji, D., Tobin, K., & Berry, V. (2012). Engaging parents in parenting programs: Lessons from research and practice. Children & Youth Services Review, 34(10), 2061-2071. https://doi.org/10.1016/j.childyouth.2012.06.011

Wrong intervention

**#2017 - Mischley 1985**
Mischley, M., Webb Stacy Jr, E., Mischley, L., & Dush, D. (1985). A parent education project for low-income families. 1985, 3(4), 45‐57.

Wrong comparator

**#97 - Fernald 2017**
Fernald, L. C. H., Kagawa, R. M. C., Knauer, H. A., Schnaas, L., García-Guerra, A., & Neufeld, L. M. (2017). Promoting child development through group-based parent support within a cash transfer program: Experimental effects on children's outcomes. Developmental Psychology, 53(2), 226-236. https://doi.org/10.1037/dev0000185

Wrong intervention

**#7 - HernandezRodriguez 2020**
Hernandez Rodriguez, J., López, C., & Moreland, A. (2020). Evaluating incentive strategies on parental engagement of the PACE parenting program. Journal of Child & Family Studies, 29(7), 1957-1969. https://doi.org/10.1007/s10826-020-01730-4

Same data as another paper

**#478 - Rostad 2017**
Rostad, W. L., Rogers, T. M., & Chaffin, M. J. (2017). The influence of concrete support on child welfare program engagement, progress, and recurrence. Children and Youth Services Review, 72, 26-33. https://doi.org/10.1016/j.childyouth.2016.10.014

Wrong intervention

**#501 - Nam 2016**
Nam, Y., Wikoff, N., & Sherraden, M. (2016). Economic intervention and parenting: A randomized experiment of statewide child development accounts. Research on Social Work Practice, 26(4), 339-349. https://doi.org/10.1177/1049731514555511

Wrong intervention

**#2019 - Bloom 1993**
Bloom, D. (1993). LEAP: Interim findings on a welfare initiative to improve school attendance among teenage parents. Ohio's Learning, Earning, and Parenting Program. 1993, (3), 22‐28.

Wrong setting

**#429 - Reininger 2018**
Reininger, T., Wyman, I., & Villalobos, C. (2018). Family trajectories and terminations in conditional cash transfer programs: The case of Chile’s Ethical Family Wage Program. Journal of Social Service Research, 44(4), 470-481. https://doi.org/10.1080/01488376.2018.1476298

Wrong intervention

**#1129 - Rahman 2018**
Rahman, M. M., & Pallikadavath, S. (2018). How much do conditional cash transfers increase the utilization of maternal and child health care services? New evidence from Janani Suraksha Yojana in India. Econ Hum Biol, 31, 164-183. https://doi.org/10.1016/j.ehb.2018.08.007

Wrong intervention

***Appendix 3: Population demographics of included studies***

|  | **Mean** | **Min** | **Max** | **Number of studies reporting** |
| --- | --- | --- | --- | --- |
| **Parent age (years)** | 32 | 29 | 38 | 6 |
| **Female parents (%)** | 62 | 0 | 93 | 5 |
| **Male children (%)** | 55 | 53 | 56 | 5 |
| **Child age (years)** | 4.9 | 3.7 | 7.7 | 5 |
| **Single Parents (%)** | 37 | 8 | 77 | 6 |
| **Ethnically and racially minoritized groups (%)** | 39 | 10 | 97 | 6 |

| ***Appendix 4.1: Risk of bias in quasi-experimental studies*** | | | | | |
| --- | --- | --- | --- | --- | --- |
|  | *Doty et al [45]* |  | *Snow et al [49]* |  | *Laxman et al [35]* |
| Similar groups | ? |  | ? |  | ? |
| Similar care in both groups | + |  | + |  | ? |
| Complete follow up | ? |  | + |  | + |
| Outcomes measured similarly | + |  | + |  | + |
| Outcomes measured reliably | + |  | + |  | + |
| Statistical analysis used | + |  | + |  | + |
|  |  |  |  |  |  |
| Nb control group irrelevant as it is an inclusion criteria, pre- and post- measures are irrelevant as outcome is attendance. “+” indicates that a feature is present, “-“ indicates a feature is absent, and “?” indicates it is not possible to determine. | | | | | |

| ***Appendix 4.2: Risk of bias in randomized controlled trials*** | | | | | | | | | |
| --- | --- | --- | --- | --- | --- | --- | --- | --- | --- |
|  | *Heinrichs (1) [48]* |  | *Heinrichs (2) [48]* |  | *Dumas et al [46]* |  | *Stanger et al [50]* |  | *Gross et al [47]* |
| True randomization | ? |  | ? |  | - |  | + |  | - |
| Concealed group allocation | ? |  | ? |  | - |  | - |  | - |
| Similar at baseline | ? |  | ? |  | - |  | - |  | + |
| Groups treated similarly | + |  | + |  | + |  | + |  | + |
| Loss to follow up | + |  | + |  | + |  | + |  | + |
| Analysed in randomized groups | + |  | + |  | + |  | + |  | + |
| Outcome measures similar | + |  | + |  | + |  | + |  | + |
| Outcome measures reliable | + |  | + |  | + |  | + |  | + |
| Deviations from standard RCT | ? |  | ? |  | + |  | + |  | + |
| Nb blinding is not possible regarding financial incentive sot it is not presented here. Failure to conceal group allocation provided useful data about the ‘connect’ stage and led to dissimilar groups. “+” indicates that a feature is present, “-“ indicates a feature is absent, and “?” indicates it is not possible to determine. | | | | | | | | | |

***Appendix 5: Parenting program attendance and financial incentives***

|  | **Incentive** | | | **Control** | | | **Completion Threshold** | **Odds Ratio** | **Hedges’ g** |
| --- | --- | --- | --- | --- | --- | --- | --- | --- | --- |
|  | **Participants** | **Passed threshold** | **Mean module completion** | **Participants** | **Passed threshold** | **Mean Module completion** |  |  |  |
| **Doty et al**** [45]** | 288 | 166 |  | 74 | 27 |  | At least one module | 1.58 |  |
| **Dumas et al[46]** | 319 | 68 | 2.6 (3.9) | 291 | 54 | 2.4 (3.8) | All modules | 1.15 | 0.05 |
| **Gross et al[47]** | 93 |  | 6.3 (4.6) | 81 |  | 5.9 (4.5) |  |  | 0.09 |
| **Heinrichs (1)[48]** | 96 | 82 | 7.5 (1.3) | 45 | 26 | 6.8 (2.3) | All modules | 1.48 | 0.41 |
| **Heinrichs (2)[48]** | 59 | 48 | 6.5 (1.9) | 48 | 31 | 6.8 (1.7) | All modules | 1.26 | -0.16 |
| **Laxman et al**** [35]** | 936 | 776 |  | 104 | 79 |  | All modules | 1.09 |  |
| **Snow et al**** [49]** | 42 | 24 |  | 37 | 23 |  | All modules | 0.92 |  |
| **Stanger et al[50]** | 29 |  | 9.4 (3.2) | 19 |  | 10.2 (3.2) |  |  | -0.25 |

**** = quasi-randomized studies.

***Appendix 6: Meta-analysis 3: Mean module completion rates among participants***

**
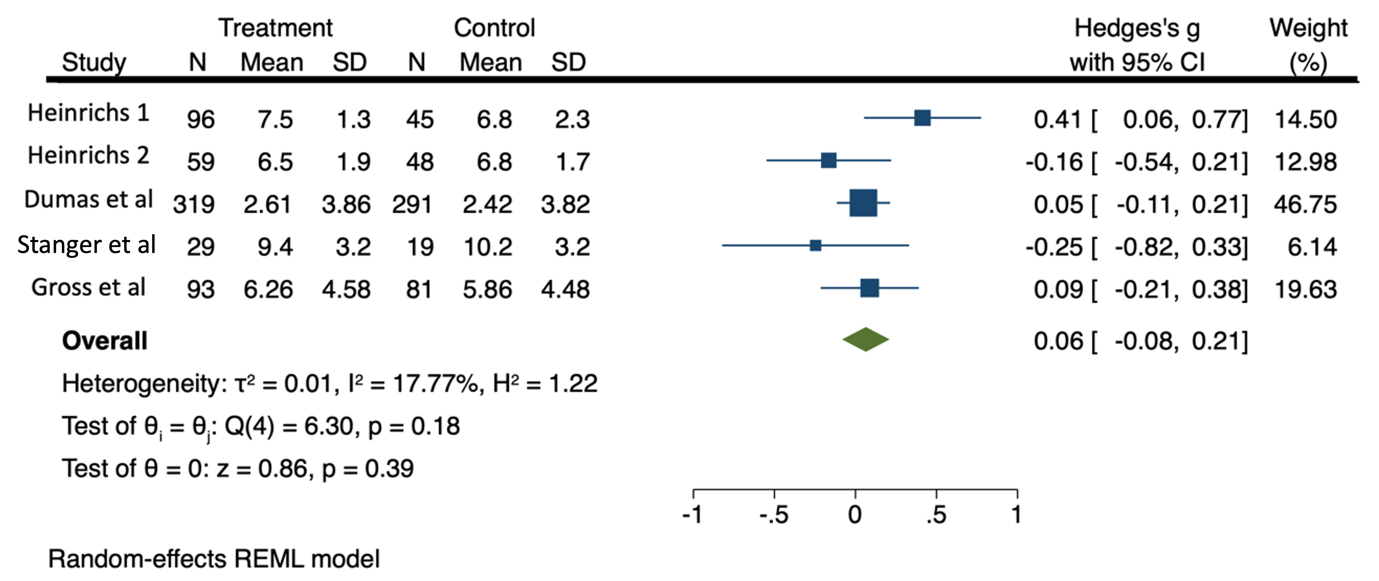
**

95% prediction interval for Hedges’ g was -0.267 - 0.395.

***Appendix 7.1: Relationship between financial incentives and proportion of participants reaching the completion threshold***

|  | **control** | | | **incentive** | | |  |  |  |
| --- | --- | --- | --- | --- | --- | --- | --- | --- | --- |
|  | **Invited** | **Participated** | **Completers** | **Invited** | **Participated** | **Completers** | **Control completers/invited** | **Incentive completers/invited** | **Log odds ratio (95% CI)** |
| Heinrichs 1 [48] | 168 | 45 | 26 | 182 | 96 | 82 | 15.5% | 45.0% | **1.07**  (0.58-1.56) |
| Heinrichs 2 [48] | 186 | 48 | 31 | 154 | 59 | 48 | 16.7% | 31.2% | **0.63**  (0.13 – 1.13) |
| Dumas et al [46] | 582 | 291 | 54 | 468 | 319 | 68 | 9.3% | 14.5% | **0.45**  (0.07 – 1.13) |

***Appendix 7.2: Meta-analysis 4: Proportions of invited parents who reached attendance threshold***

**
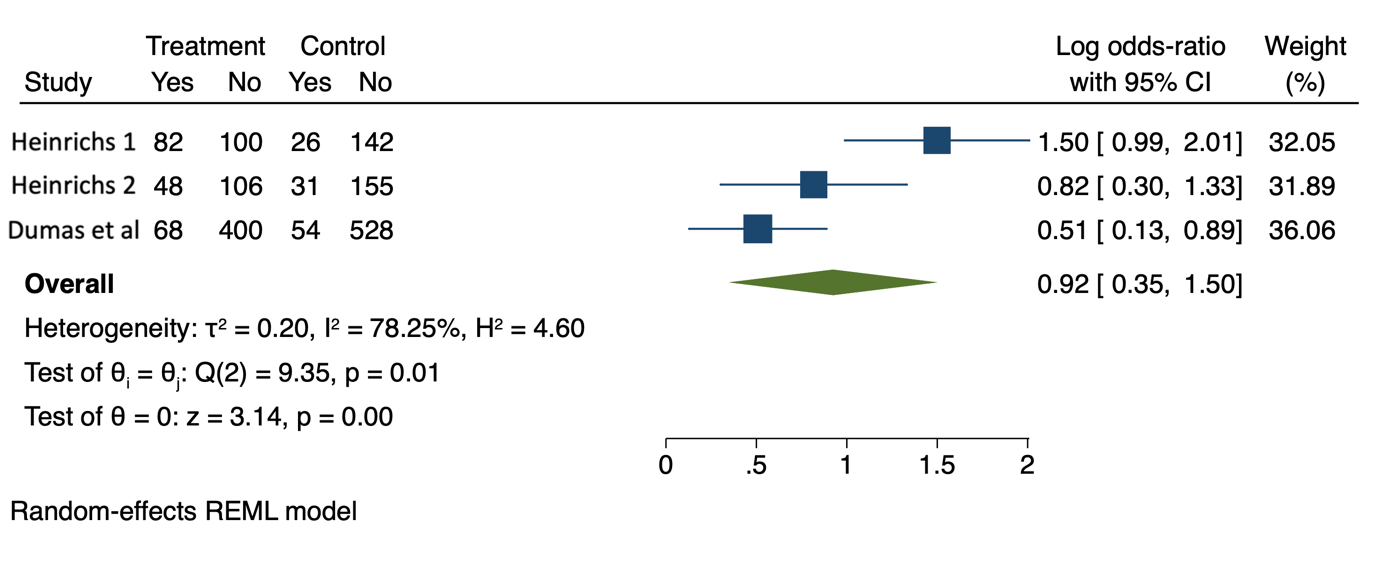
**

The 95% prediction interval for the OR was -5.91 - 2344.90.

**Appendix 8: How data from each included study fits the CAPE framework**

|  | | Doty et al | | Dumas et al | | Gross et al | | Heinrichs 1 | | Heinrichs 2 | | Laxman et al | | Snow et al | | Stranger et al | |
| --- | --- | --- | --- | --- | --- | --- | --- | --- | --- | --- | --- | --- | --- | --- | --- | --- | --- |
|  |  | Ix | Ctrl | Ix | Ctrl | Ix | Ctrl | Ix | Ctrl | Ix | Ctrl | Ix | Ctrl | Ix | Ctrl | Ix | Ctrl |
| **Connection** | Attended | - | - | 96 v 182 | 45 vs 168 | 93 vs 395 | 81 v 397 | 59 v 154 | 48 v 186 | 319 v 468 | 291 v 582 | - | - | - | - | - | - |
| **Attend** | Threshold | 166 v 288 | 27 v 74 | - | - | - | - | 82 v 96 | 26 v 45 | 48 v 59 | 31 v 48 | 776 v 936 | 79 v 104 | 24 v 42 | 23 v 37 | - | - |
|  | Mean modules completed | - | - | 2.6 (3.9) | 2.4 (3.8) | 6.3 (4.6) | 5.9 (4.5) | 7.5 (1.3) | 6.8 (2.3) | 6.5 (1.9) | 6.8 (1.7) | - | - | - | - | 9.5 (3.2) | 10.2 (3.2) |
| **Participate** | Homework exercises completed | - | - | - | - | - | - | - | - | - | - | - | - | - | - | 41% | 21% |
| **Engage** | (No measures reported) | - | - | - | - | - | - | - | - | - | - | - | - | - | - | - | - |

**Appendixes 9.1 and 9.2: Funnel plots showing the relationship between standard error and engagement outcomes**

**Appendix 9: GRADE Analysis explanations**

Finding 1 is based on meta-analysis of 4 RCTs with over 1000 participants and supported by meta-analyses 1 and 2. There is very low risk of bias. There is moderate heterogeneity. There is no inconsistency of effect direction. Measurement is not indirect and there is no evidence of publication bias. High certainty.

Finding 2 is based on meta-analysis of 4 studies with over 2000 participants. All are RCTs. Risk of bias is very low. Heterogeneity is low. There is no inconsistency of effect direction. The CI is narrow. Measurement is not indirect and there is no evidence of in publication bias. High certainty.

Finding 3 is based on a meta-analysis of 6 studies with over 1000 participants. Four are RCTs and two are quasi-experimental. Low risk of bias. Very low heterogeneity. There is some heterogeneity of effect direction. Measurement is not indirect and there is no evidence of in publication bias. Moderate certainty.

Finding 4 draws on three studies including over 2000 invited people. Two were RCTs with low risk of bias. One was a quasi-experimental study which is at greater risk of bias. Measurement is not indirect and there is no evidence of in publication bias. There is inconsistency in exact demographic differences and in effect magnitude but no inconsistency in effect direction. Moderate certainty.

Finding 5 also draws on three studies including over 2000 invited people. Two were RCTs with low risk of bias. One was a quasi-experimental study which is at greater risk of bias. Measurement is not indirect and there is no evidence of in publication bias. There is inconsistency in measurement method and in effect magnitude but no inconsistency in effect direction. Moderate certainty.

Finding 6 is based on subsequent pooled data from two of the included RCTs plus another RCT including over 200 participants. Risk of bias is low. No meta-analysis was possible. One father-rated score conflicts but is in general treated as less informative than mother-rated score. Measurement is not indirect and there is no evidence of in publication bias. Low certainty.

Finding 7 is an attempt to reconcile the descriptions of all the incentive designs with the results regarding engagement. It draws on all 8 studies including 6 RCTs and over 2000 participants. Within the data the pattern is clear, but it was not a prespecified hypothesis in the protocol, creating a risk of bias in analysis. Measurement is indirect but there is no evidence of in publication bias. There is inconsistency in measurement method but the effect direction appears consistent. Low certainty.
