# Supplementary material for: Review: Systematic review and meta‐analysis – financial incentives increase engagement with parenting programs for disruptive behavior problems
Source: Child Adolesc Ment Health. 2024 Dec 21;30(1):53–65. doi: 10.1111/camh.12746 (PMC11754718; doi:10.1111/camh.12746)
Supplement: Supplementary file 2 — Appendix S10. Full GRADE table. [file CAMH-30-53-s002.docx]

**Appendix S10: Full GRADE Table**

| **Outcome** | **Relative Effect (95% CI)** | **Relative Effect (95% CI)** | **Anticipated absolute effects** | | | **Number of participants (number of studies)** | **Certainty of the Evidence (GRADE)** | **Comments** |
| --- | --- | --- | --- | --- | --- | --- | --- | --- |
|  |  |  | **Engagement with incentives** | **Engagement without incentives** | **Difference** |  |  |  |
| People invited to incentive group are more likely to reach threshold number of sessions. | Log odds ratio 0.69 (0.33-1.06). | Odds ratio 2.00 (1.39-2.88) | 19.8% | 10.6% | 10.2 percentage points | 2049  (3 or 8) | High  (+)(+)(+)(+) | 3 studies in the main meta-analysis showed a moderately sized significant correlation, and 8 studies across meta-analyses 3 and 4 also provided supporting evidence |
| More parents connect with parenting programs when they know there will be incentives for participation. | Log odds ratio 0.34 (0.18-0.50) | Odds ratio 1.40  (1.20-1.65) | 32.1% | 25.9% | 7.2 percentage points | 3564  (4) | Moderate  (+)(+)(+)(-) | Across 4 studies there was a small significant correlation |
| Incentives increase how many parents reach threshold number of sessions. | Log odds ratio 0.57, (0.16 – 0.98) | Odds ratio 1.76 (95% CI: 1.17 – 2.66 | 40.1% | 28.6% | 11.5 percentage points | 3743  (6) | Moderate (+)(+)(+)(-) | Across 6 studies there was a small significant correlation |
| Incentives increase connection among people with lower incomes. | n/a | n/a | n/a | n/a | n/a | (3) | Moderate (+)(+)(+)(-) | Three studies reported consistent findings from different measurements. |
| Incentives increase connection among ethnically and racially minoritized groups as well as immigrant populations. | n/a | n/a | n/a | n/a | n/a | (3) | Moderate (+)(+)(+)(-) | Three studies reported inconsistent results and used different measurements |
| Evidence suggests no difference in behaviour change between incentive and control groups. | n/a | n/a | n/a | n/a | n/a | (3) | Low (+)(+)(-)(-)  Due to indirect measurement | Conflicting results across three studies. |
| Guaranteed incentives for each attendance valued over $10 are most effective at increasing engagement. | n/a | n/a | n/a | n/a | n/a | (8) | Low  (+)(+)(-)(-)  Due to lack of data | Only one study directly compared two measures |
